# The Clock Keeps on Ticking: Age and Sex Outcomes for Acute Type A Aortic Dissection∗

**DOI:** 10.1016/j.jacadv.2024.100911

**Published:** 2024-04-24

**Authors:** Joseph C. Cleveland, Michael Cain

**Affiliations:** Division of Cardiothoracic Surgery, Department of Surgery, University of Colorado Anschutz Medical Center, Aurora, Colorado, USA

**Keywords:** aortic aneurysm, aortic disease, sex

**Figure:**
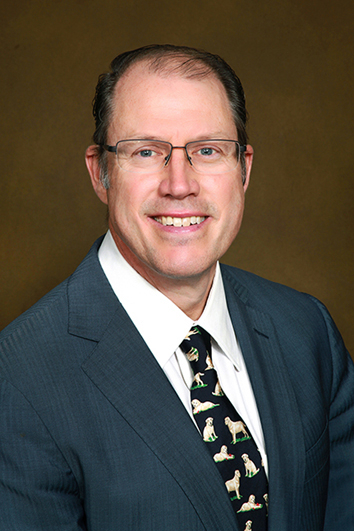

The mortality and morbidity of acute type A aortic dissection remain significant despite many advances in the surgical treatment of this disorder.^1^ Many factors account for the observed 10% mortality reported in most contemporary series: delay in diagnosis, myocardial ischemia, and malperfusion with circulatory shock are among the leading causes of mortality. In this issue of *JACC: Advances*, Liu et al^2^ representing the 5A investigators explore the interaction of age and sex on short-term mortality after type A aortic dissection.

The current study has many strengths; it represents a large multicenter registry of patients undergoing surgery for acute type A aortic dissection in China. Noteworthy is that this analysis included propensity matching for 1,002 patients to adjust for differences in baseline characteristics. The surgical techniques employed in the study are state-of-the-art, which is reflected in the total arch replacement treatment for 60% of the entire study cohort. The data indeed gives seemingly conflicting results. The unadjusted operative mortality is higher for women than men. This difference appears to disappear with propensity matching. Finally, when sophisticated analyses examine the interactions of age and sex, there appears to be a signal of worse operative mortality in females >55 years of age.

What new lessons have we learned from this analysis? There are prior registry data suggesting that age impacts operative mortality after type A aortic dissection. However, the age association with worsened outcomes generally appears at least a decade later, usually in patients older than 70 years of age. The current study highlights that female sex interacts with age. Women have generally had worse outcomes than men after type A aortic repair; however, in the IRAD (International Registry of Aortic Dissection) registry, women tend to be older than men at presentation. Thus, is it sex alone or sex and increasing age that matter?

Type A aortic dissection remains a highly heterogenous disease process. Thus, important interactions between age and sex are clearly operant. The current study elegantly examines this interplay and suggests a relationship exists between older age and female sex in producing worse outcomes. However, the findings of this current observational study still leave much open to discussion. Should surgical management differ between older females and younger males? How could screening programs more accurately identify these elderly female populations at risk and intervene on most of these aneurysmal patients before they present with a life-threatening catastrophic type A dissection? This study highlights the continued opportunity to enhance our understanding of type A aortic dissection.

## Funding support and author disclosures

Dr Cleveland, Jr has relationships with Abbott Medical and Medtronic. Dr Cain has reported that he has no relationships relevant to the contents of this paper to disclose.
